# Differential tissue tropism and transmission efficiency of two dominant influenza D clades with overlapping but distinct receptor binding fine specificities in ferrets

**DOI:** 10.1371/journal.ppat.1013493

**Published:** 2025-09-15

**Authors:** Tirth Uprety, Chithra C. Sreenivasan, Jieshi Yu, Miaoyun Zhao, Runxia Liu, Hai Yu, Ahsan Naveed, Lianne G. Eertink, Shalini Soni, Rebecca E. Ruby, Xi Chen, Radhey S. Kaushik, Zizhang Sheng, Qingsheng Li, Dan Wang, Feng Li

**Affiliations:** 1 Department of Veterinary Science, M. H. Gluck Equine Research Center, University of Kentucky, Lexington, Kentucky, United States of America; 2 Nebraska Center for Virology, University of Nebraska-Lincoln, Lincoln, Nebraska, United States of America; 3 School of Biological Sciences, University of Nebraska-Lincoln, Lincoln, Nebraska, United States of America; 4 Department of Biology and Microbiology, South Dakota State University, Brookings, South Dakota, United States of America; 5 Department of Chemistry, University of California, Davis, California, United States of America; 6 Department of Veterinary Science, Veterinary Diagnostic Laboratory, University of Kentucky, Lexington, Kentucky, United States of America; 7 Aaron Diamond AIDS Research Center, Columbia University Vagelos College of Physicians and Surgeons, New York, United States of America; 8 Division of Infectious Diseases, Department of Medicine, Columbia University Vagelos College of Physicians and Surgeons, New York, United States of America; Texas Biomedical Research Institute, UNITED STATES OF AMERICA

## Abstract

Influenza D virus (IDV) utilizes bovines as a primary reservoir causing periodical spillover to pigs and other hosts. In this study, we utilized ferrets to study IDV with a focus on the role of the Hemagglutinin-Esterase-Fusion (HEF) protein in the replication, tissue tropism, and transmission of two dominant clades of IDV– swine D/OK, and bovine D/660. In addition to swine D/OK, we rescued a chimeric virus (D/OK^660HEF^) expressing the bovine D/660 HEF using reverse genetic system. Two isogenic IDVs differing only in the HEF protein were characterized in ferrets with respect to viral shedding, tissue tropism, transmission, and pathogenesis. Ferrets intranasally infected with D/OK and D/OK^660HEF^ showed similar levels of viral shedding but exhibited slight differences in transmission efficiency to contact sentinel ferrets. Specifically, D/OK replicated mostly in the upper respiratory tract and transmitted to 2/3 naive ferrets, while D/OK^660HEF^ replicated in both upper and lower respiratory tract (trachea) but transmitted only to 1/3 naive ferrets. Both direct inoculated and contact sentinel ferrets seroconverted at 14 days post-infection, which indicated an association with viral replication fitness and transmission efficiency. Distinct receptor fine specificities plus six amino acid mutations in the receptor binding domain of the HEF protein between swine D/OK and bovine D/660 viruses may explain the different tissue tropism and transmission efficiency observed between these two viruses. Furthermore, while no detectable virus titers were observed in the lungs and intestines of ferrets, fluorescent RNAscope probe-based *in-situ* hybridization assay detected viral RNAs in these tissues. Finally, deep-sequencing revealed ferret-adapted mutations in PB1, PB2, and M segments that have not appeared in natural IDV isolates from bovines or pigs which need further characterization. Taken together, results of this study demonstrate that IDV is optimized for replication and spread in mammals and subtle mutations in HEF protein may affect viral tropism and transmission efficiency.

## Author summary

Although no human infection is reported, genome fragments of bovine influenza D virus and its antibodies were detected in humans. To evaluate the zoonotic potential of IDV and to gain a deeper understanding of the *in vivo* function of the viral hemagglutinin-esterase-fusion (HEF) protein, we generated two isogenic IDVs (D/OK and D/OK^660HEF^) that varies only in the HEF protein, which represents two major IDV clades (D/OK and D/660). We then characterized these viruses in ferrets for viral shedding, tissue tropism, transmission, and pathogenesis. We show that both WT D/OK and D/OK^660HEF^ replicate and shed in intranasally inoculated ferrets and can transmit the virus to contact sentinel ferrets. WT D/OK appeared to replicate primarily in the upper respiratory tract and was associated with higher transmissibility, whereas D/OK^660HEF^ showed replication in both the upper and lower respiratory tracts and tended to exhibit lower transmissibility. This finding is further confirmed by the seroconversion data. Furthermore, we reveal that the distinct receptor fine specificities previously observed between swine D/OK and bovine D/660 viruses may be attributed to six amino acid mutations in the receptor binding domain of the HEF between the two viruses. Additionally, we notice several ferret-adapted mutations in PB1, PB2, and M segments of IDV.

## Introduction

In 2011, a novel influenza virus was isolated from a diseased piglet exhibiting influenza like symptoms [1]. Further genetic and antigenic characterization led to its classification as a new species (type), influenza D, in the family *Orthomyxoviridae* [2,3]. At present, the *orthomyxoviridae* has seven genera: *Alphainfluenza virus, Betainfluenza virus, Gammainfluenza virus, Deltainfluenza virus, Isavirus, Thogotovirus,* and *Quaranjavirus* [4]. The first four influenza genera have single species namely influenza A (IAV), influenza B (IBV), influenza C (ICV), and influenza D (IDV) virus, respectively.

Influenza viruses are significant health concerns for humans. While IAVs have caused numerous human pandemics [5,6], IBV infections are limited to seasonal epidemics [7]. ICV, which is more closely related to IDV among influenza viruses, primarily cause mild infections in humans [8–10], however, ICV has been detected in cattle with mild to moderate respiratory illness lately [11–14]. In recent years, ICV has been associated with cases of severe pediatric respiratory infections [15–17] and the associated illness can be more severe in children younger than two years old. Despite the IDV that was first identified in swine, cattle are a primary reservoir of IDV. IDV causes mild respiratory illness in swine and cattle [1,2] with occasional spillover to other farm animals like sheep, goats, horses, and camels, [18–22]. In addition to domestic farm species, several studies have confirmed the presence of IDV antibodies in wildlife like wild boar, deer, and hedgehogs [20,23]. Thus, IDV has a broad host tropism similar to IAV. So far no human infection with IDV has been reported, although antibodies against IDV were detected in humans, and viral RNA genome fragments were found in nasal washes of cattle handlers [1,24–26] as well as of swine farmers [27]. Furthermore, IDV genomes were also detected in bio-aerosol samples collected from airport [28] and hospital emergency room [29]. This data combined with the evidence of the presence of IDV-specific antibodies in general population with no prior history of occupational exposure [1] indicate that the non-agricultural workers might also get exposed to IDV. Thus IDV has the potential to emerge as a human pathogen, which is further supported by the fact that it selectively uses 9-*O*-acetylated *N*-acetylneuraminic acid (Neu5,9Ac_2_) abundantly expressed in humans, and 9-*O*-acetylated *N*-glycolylneuraminic acid (Neu5Gc9Ac) abundantly expressed in agricultural animals as functional entry receptor in *in-vitro* assays [30,31]. In addition, the recent emergence of highly pathogenic avian influenza H5N1 in bovine, transmission across dairy cattle herds, and spillover into humans warrants further investigation of bovine IDV and its widespread transmission in the global cattle population consisting of beef and dairy cows [32].

Despite IDV’s potential to emerge as a human pathogen, very few studies have been conducted in the context of a human relevant model for risk assessment of IDV to human health in terms of replication fitness, transmission, and pathogenicity. Small animal models like guinea pigs, mice, or even large animal models such as swine and cattle do not exactly recapitulate influenza infection and transmission in humans, as sialic acid species and distribution patterns in these animals are different from humans [33]. For example, *N*-acetylneuraminic acid (Neu5Ac) and *N*-glycolylneuraminic acid (Neu5Gc) are two major forms of sialic acids in mammals [34]. Neu5Gc is introduced at the sugar nucleotide stage by CMP-Neu5Ac hydroxylase (CMAH)-catalyzed hydroxylation of cytidine 5’-monophosphate (CMP)-Neu5Ac to CMP-Neu5Gc [35], which is then used as a donor substrate for sialyltransferases to form Neu5Gc-containing glycans and glycoconjugates. The conversion of Neu5Ac to Neu5Gc is a unidirectional process. Humans have a dysfunctional CMP-Neu5Ac hydroxylase (CMAH) so Neu5Gc and its derivatives are not expressed in humans [36]. In contrast, agricultural animals including swine, cattle, equine, and small ruminants have a functional *CMAH* gene, expressing higher levels of Neu5Gc on the epithelial surface along their respiratory and gastrointestinal tracts [37]. Among small animal models, ferrets are unique with a non-functional *CMAH* gene, so Neu5Gc is absent in this animal species, which is similar to humans [38]. In addition, ferrets resemble humans in lung physiology [38,39] and the binding pattern of avian and human IAVs in the respiratory tract of ferrets is congruent to that in human respiratory tract [40]. Porcine Torovirus (PToV) based probe based binding assay indicated that like humans, ferret have comparable levels of the 9-*O*-acetyl and 7,9- *O*-acetyl modified sialic acid [33]. These features support the utility of ferrets as an exceptional animal model for human influenza studies. With exceptions of some highly pathogenic influenza A strains (pandemic H1N1 2009, H5N1, H7N7, H7N9), influenza viruses do not readily infect mice without adaptation mutations [41]. While guinea pigs do not need prior adaptation, they do not recapitulate many disease symptoms that are observed in human infections, and they are different from humans in sialic acid forms and distribution [33,42,43]. Therefore, ferrets are commonly used as a surrogate model for rapid assessment of pandemic or zoonotic risk posed by animal influenza viruses [40,42,44,45].

The prototypic strains D/swine/Oklahoma/1334/2011 (Swine D/OK) and D/Bovine/Oklahoma/660/2013 (bovine D/660), both originated subsequently from Oklahoma Panhandle region, are commonly used as representative strains of two dominant phylogenetic clades of IDVs: D/OK and D/660 [46,47], based on the nucleotide sequence encoding the HEF protein, the primary antigenic determinant of IDV. An early study showed that swine D/OK can infect ferrets and transmit to naive ferrets by direct contact, indicating its potential zoonosis [1]. Despite progress, the replication dynamics, tissue tropism, and pathogenesis are still poorly characterized for swine D/OK in ferrets. IDV and its closely related ICV differ from IAV and IBV in that they only express one major surface glycoprotein (HEF) [48,49]. The HEF protein performs all entry functions including receptor binding, receptor destroying, and fusion [48,50,51]. Bovine D/660 belongs to the IDV D/660 clade that is distinct from swine D/OK in antigenic property [46,47]. Bovine IDVs including D/660 have not been studied in ferrets yet for zoonotic risk assessment. HEF-dependent replication dynamics, tissue tropism, and transmission efficiency in ferrets are not known for bovine D/660 and its related bovine IDVs. In this study, we leveraged on the swine D/OK’s reverse genetics system [52] recently developed in our laboratory and generated a chimeric virus (D/OK^660HEF^) in that bovine D/660 HEF segment substituted its counterpart in the context of the swine D/OK backbone. Two viruses (D/OK and D/OK^660HEF^) that varied only in HEF protein were fully examined and compared in ferrets towards elucidating the role of HEF protein in replication kinetics, tissue tropism and pathogenesis, and transmission efficiency of two dominant IDV clades in ferrets.

## Results

### Replication fitness and transmission kinetics of swine D/OK and D/OK^660HEF^ (variant expressing bovine D/660 HEF) in ferrets

**Fig 1:**
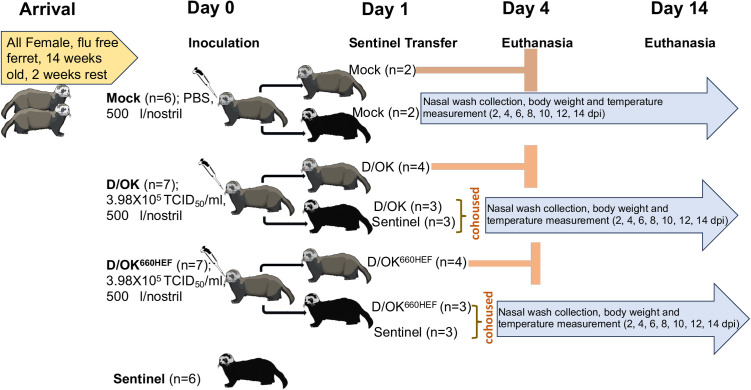
Schematic representation of experimental design adopted in this study. Influenza virus antibody-negative ferrets, after 2 weeks of acclimatization, were either intranasally inoculated with 3.98 x 10^5^ TCID_50_/ml of the virus, 500 μl/nostril of D/OK (n = 7), or D/OK^660HEF^ (n = 7) virus at day 0. At day 0, four ferrets were also mock inoculated with sterile 1X PBS (500 μl/nostril) (n = 4), and sentinel ferrets (n = 6) received neither the virus nor the mock PBS. One day post inoculation, 6 sentinel ferrets were split into two groups of 3 each and were cohoused with swine D/OK (n = 3), or bovine DOK^660HEF^ (n = 3) intranasally inoculated ferret in 1:1 donor to receiver ratio. These ferrets constituted the transmission study group, which also included two mock ferrets. Nasal washes were collected every other day from transmission study group and all remaining ferrets were euthanized on day 14 of the experiment. Four of the seven challenged ferrets that were not part of transmission study (for both D/OK and D/OK^660HEF^) were euthanized at 4-day post infection, including 2 mocks. The bio-art used in this figure were obtained from NIAID Visual & Medical Arts available in public domains (NIAID Visual & Medical Arts. bioart.niaid.nih.gov/bioart/150).

To determine and compare the HEF-dependent replication kinetics of IDV in ferrets, three ferrets per group were intranasally inoculated with 3.98 x 10^5^ TCID_50_ of swine D/OK and its variant expressing bovine D/660 HEF segment, respectively, while two ferrets were mock infected with PBS as controls for this experiment (Fig 1). Infected and control ferrets were observed daily for clinical signs including coughing, sneezing, labored breathing, and nasal discharges. Body weight and temperatures were also recorded for each animal during a period of 14 days post infection (dpi). Neither clinical signs nor changes in body weight and temperature were observed for infected ferrets between two groups (S1 Data). Overall, the infected ferrets behaved similarly to PBS mock infected animals.

**Fig 2:**
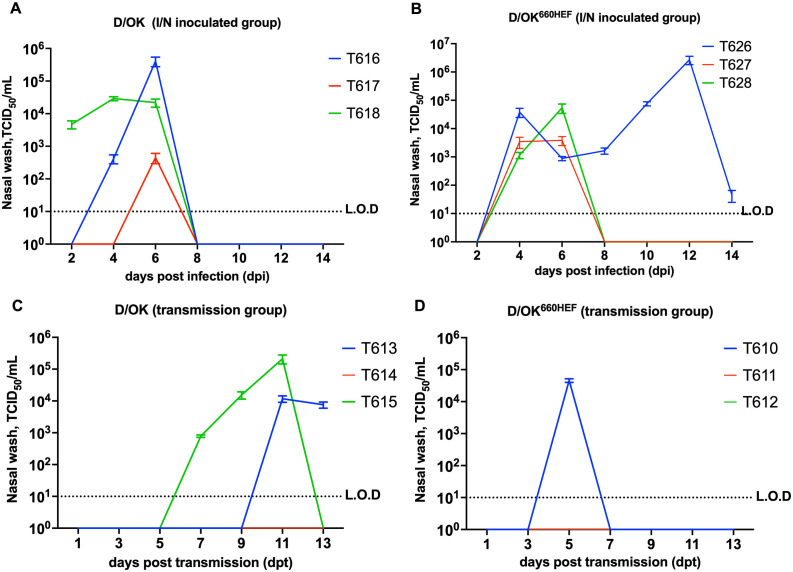
Viral shedding in nasal washes of intranasal inoculated and sentinel ferrets in transmission groups. Nasal washes were collected every other day and were titrated with the standard TCID_50_ assay. (A) Viral shedding in nasal washes of ferrets inoculated with D/OK virus (ferret ID: T616, T617, and T618). (B) Viral shedding in nasal washes of ferrets inoculated with D/OK^660HEF^ virus (ferret ID: T626, T627, T628). (C) Viral shedding in contact sentinel ferrets, cohoused with D/OK inoculated ferrets (ferret ID: T613, T614, T615). (D) Viral shedding in contact sentinel ferrets, cohoused D/OK^660HEF^ inoculated ferrets (ferret ID: T610, T611, T612). Note that the color scheme of the kinetics graph in D/OK or D/OK^660HEF^ intranasal inoculated and transmission group indicates that ferrets indicated with the same color were cohoused together. Mock ferrets (T608, and T609) were negative for the virus.

Viral shedding was determined by the quantitative measurement of virus loads in nasal washes, which were collected from the directly inoculated and mock-infected ferrets at 48-h intervals from day 1 through day 14 post-infection (dpi). As summarized in Fig 2., viral shedding was observed in all three ferrets in both groups. In swine D/OK intranasally (IN) infected ferrets, virus shedding was observed as early as 2 dpi and virus clearance occurred by 8 dpi (Fig 2A). All three ferrets showed virus shedding throughout 6 dpi, with ferret T616 reaching the highest viral load (5.5 log TCID_50_/mL) at 6 dpi among swine D/OK infected ferrets. In three ferrets infected with D/OK^660HEF^, virus shedding in nasal washes was observed in all three of them and peak titers were comparable with those observed in swine D/OK infected animals (Fig 2A and 2B). For all D/OK^660HEF^ intranasally (IN) infected ferret, virus shedding started at 4 dpi and discontinued by 8 dpi in 2/3 ferrets. One of the ferrets showed prolonged virus shedding till 14 dpi with the peak viral titer observed at 12 dpi (6.3 log TCID_50_/mL). The other two ferrets that had viral clearance by 6 dpi showed peak viral titers of 4.6 log TCID_50_/mL and 3.4 log TCID_50_/mL at 6 dpi, respectively (Fig 2B). Taken together, both swine D/OK and D/OK^660HEF^ replicated in the respiratory tract of ferrets. Although bovine D/660 HEF and swine D/OK HEF exhibited similar replication kinetics, ferrets inoculated with the chimeric D/OK^660HEF^ virus showed a trend toward increased viral titers over time compared to those infected with swine D/OK. Notably, the mean viral titers at 4 and 6 dpi were approximately 10-fold higher in the D/OK^660HEF^ group. While this observation suggests a potential replication advantage conferred by the bovine HEF, further statistical analyses with larger sample size are needed to confirm this trend. In addition, these findings need to be corroborated with bovine D660 virus containing a HEF protein from swine D/OK to further strengthen our conclusion that IDV replication differences is driven by lineage specific HEF protein.

Next, we tested whether HEF protein plays a role in transmission efficiency of IDV in ferrets by taking advantage of swine D/OK and its D/OK^660HEF^ variant that differs only in HEF protein. Three intranasally inoculated ferrets in each group as described above were cohoused at 1 dpi with three sentinel animals with one infected and one native animal housed in the same cage. This experimental setup enabled simultaneous detection of the transmission events mediated through direct contact and through respiratory droplets/aerosols in the absence of direct contact. Clinical signs including changes in the body weight and temperature of naïve ferrets cohoused with infected animals were monitored daily and the transmission efficiency of the virus in ferrets through contact was measured by quantitatively analyzing viral loads in the nasal washes, nasal turbinate, and lungs from the infected and sentinel animals over a period of 14 days. Nasal washes were collected at 48-h intervals from 2 dpi to 14 dpi, and all the animals were euthanized at 14 dpi. In swine D/OK transmission group, two out of three native ferrets got infected after exposure to directly infected ferrets. Transmission of swine D/OK to ferret T615 from directly inoculated ferret T618 began at 7^th^ day post ferrets co-mingling (Fig 2C). Viral titers in ferret T615 continued to increase over time, reaching peak viral load (5.1 log TCID_50_/ml) at 11 dpt (day post transmission) and viral shedding discontinued at 13 dpt. Swine D/OK transmission to ferret T613 from inoculated ferret T616 was not detected until 11^th^ day post ferret cohousing but continued to shed at 13 dpt. Compared to swine D/OK transmission group, only one (T610) of three naïve ferrets got infected when cohoused with directly inoculated ferrets with D/OK^660HEF^ variant with bovine HEF (Fig 2D). Despite shedding a high titer (4.6 log TCID_50_/mL) at 5 dpt, this ferret (T610) had a very short duration of viral shedding in that viral replication was only detected at 5 dpt, during the 14-day period (Fig 2D). The observed differences in duration of viral shedding and the number of infected cohoused ferrets may suggest a potential difference in transmission dynamics between swine D/OK and bovine D/660 driven by the HEF protein. However, any inference regarding the enhanced transmission efficiency should be interpreted in light with the limited sample size and absence of transmission data from other control viruses such as bovine D/660 expressing swine D/OK HEF protein.

### Tissue tropism of swine D/OK and D/OK^660HEF^ variant expressing bovine D/660 HEF in ferrets

**Fig 3:**
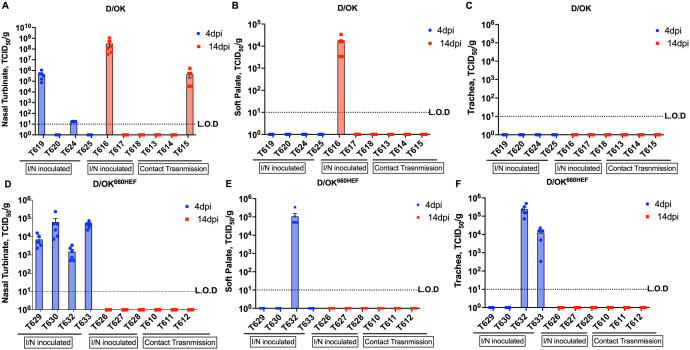
Tissue tropism of D/OK and D/OK^660HEF^ viruses. Ferrets were euthanized at 4 dpi, or 14 dpi and tissues were collected respectively. The standard TCID_50_ assay was performed on tissue after weighing and homogenization. Viral titers were normalized per gram of tissue. Viral titers in tissue from ferrets euthanized at 4 dpi are shown in blue bars, while the orange bars represent tissues from ferrets euthanized at 14 dpi. Detection of viable virus in nasal turbinate (A), soft palate (B), and trachea (C) in D/OK inoculated ferrets euthanized at 4 dpi (T619, T620, T624, T625) or 14 dpi (T616, T617, T618), and contact sentinel ferrets euthanized at 13 dpt (T613, T614, T615). Detection of viable virus in nasal turbinate (D), soft palate (E), and trachea (F) in D/OK^660HEF^ inoculated ferrets euthanized at 4 dpi (T629, T630, T632, T633) or 14 dpi (T626, T627, T628), and contact sentinel ferrets euthanized at 13 dpt (T610, T611, T612). Tissues obtained from two mocks euthanized at 4 dpi (T605 and T607) showed no detectable virus.

Tissue tropism of swine D/OK and its variant D/OK^660HEF^ in ferrets was investigated and compared by identifying the sites of viral replication using tissue homogenates collected either at 4 dpi, 14 dpi, or 13 dpt. Four ferrets inoculated with swine D/OK and euthanized at 4 dpi showed that two of four ferrets (2/4 ferrets) exhibited detectable viral replication in nasal turbinate (Fig 3A). In. intranasal D/OK inoculated ferret euthanized at 14 dpi, one of three ferrets (1/3 ferrets) demonstrated viable viruses in soft palate (Fig 3B). Mean virus titer was higher in nasal turbinate (5.5 log TCID_50_/gram) than those in soft palate (4 log TCID_50_/gram). No viable viruses were found in trachea (Fig 3C) and lungs at 4 dpi. In ferrets, euthanized at 14 dpi, one of three ferrets (1/3 ferrets) in both swine D/OK inoculation and contact transmission groups had active viral replication in nasal turbinate (Fig 3A). A higher viral load was detected in the nasal turbinate at 14 dpi (8.1 log TCID_50_/gram) in swine D/OK inoculated ferret than that in the nasal turbinate (5.1 log TCID_50_/gram) of the contact transmission ferret (Fig 3A). Interestingly, these two ferrets were cohoused together as D/OK inoculated (T618) and contact sentinel (T615) ferrets (Fig 2A and 2C). It should be noted that detection of active viral replication in the nasal turbinate of a contact ferret (T615) further supports the transmission data as measured in viral shedding experiment (Fig 2C) for this animal. Furthermore, no viable virus was detected in the trachea, lungs, intestine, and fecal swabs for all of the ferrets in swine D/OK intranasally inoculated group or swine D/OK contact transmission group. These data appeared to suggest that swine D/OK HEF confines IDV to replicate within the upper respiratory tract of ferrets (nasal turbinate and soft palate).

In four ferrets that were inoculated with D/OK^660HEF^ variant expressing bovine HEF and euthanized at 4 dpi, infectious viruses were detected in nasal turbinate of all four ferrets (Fig 3D), unlike swine D/OK inoculation group in which only 2/4 ferrets showed detectable viral replication in this upper respiratory tissue (Fig 3A). Mean viral titers ranged from 3 log TCID_50_/gram to 4.6 log TCID_50_/gram in these D/OK^660HEF^ infected ferrets. Furthermore, at 4 dpi, viable viruses were found in the soft palate in one of four ferrets (1/4 ferrets), with mean viral titer of 5.3 log TCID_50_/gram (Fig 3E). Interestingly, two of four D/OK^660HEF^ infected ferrets showed the active virus replication in the trachea at 4 dpi (Fig 3F), which further separated D/OK^660HEF^ inoculation group from swine D/OK group in that no infectious viruses were found in trachea of swine D/OK infected ferrets. Mean viral titers in trachea were 3.8 log TCID_50_/gram to 5.3 log TCID_50_/gram. Two ferrets (T632 and T633) inoculated with D/OK^660HEF^ variant demonstrated viable viruses in two or three tissues: nasal turbinate, soft palate, and trachea (Fig 3D - 3F).

In both D/OK^660HEF^ variant inoculated and contact transmission groups, no viable viruses were detected in nasal turbinate, soft palate, or trachea at 14 dpi (Fig 3D - 3F). Similar to swine D/OK group, no viable replication in lungs, intestine, and fecal swabs was detected in ferrets euthanized at 4 dpi or 14 dpi in D/OK^660HEF^ group. Although the results are inferred from small sample size, the D/OK^660HEF^ variant expressing bovine HEF exhibited slightly lower transmission efficiency in ferrets compared to the swine D/OK strain, D/OK^660HEF^ appeared to replicate slightly efficiently in the ferret lower respiratory tract, extending into the trachea. These findings may indicate that bovine HEF could facilitate viral access to the lower respiratory tract for replication, although it may be less effective than swine HEF in enhancing the transmissibility in this model.

### Seroconversion in ferrets after experimental inoculation and transmission

**Fig 4:**
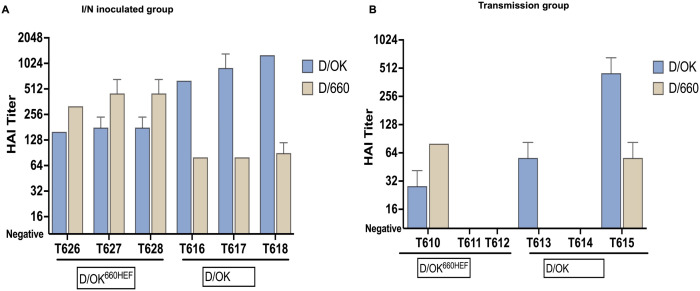
Seroconversion in intranasal inoculated and contact sentinel ferrets using hemagglutination inhibition assay (HAI). Serial two-fold dilution of ferret sera (starting from 1:10 dilution) were incubated for half an hour at room temperature with 4 HA units of swine D/OK or bovine D/660 virus. Virus neutralization titers were measured by inhibition of binding to 1% Turkey RBC. HAI titers were expressed as reciprocal of highest dilution that showed complete absence of agglutination. (A) Seroconversion and HAI titers in D/OK (T616, T617, T618) and D/OK^660HEF^ (T626, T627, T628) intranasal inoculated ferret groups. (B) Seroconversion and HAI titers of contact sentinel ferrets co-housed with D/OK (T613, T614, T615) or D/OK^660HEF^ intranasal inoculated ferrets (T610, T611, T612). Experiment was performed in duplicate and repeated thrice. Value represents geometric mean with error bar representing geometric standard deviation.

To assess serum antibody response to swine D/OK and D/OK^660HEF^ variant in ferrets, we collected blood before and after -inoculation (4 and 14 dpi or dpt) and assessed antibody titers by HI assay. Blood samples from mock infected ferrets are also used as controls in HI assay. All pre-inoculation sera, mock sera, as well as 4 dpi sera in both groups were negative for IDV antibodies in HI assay. As shown in Fig 4, all three ferrets inoculated with swine D/OK virus seroconverted at 14 dpi. The homologous geometric mean HI titers (GMT) were 640, 905, and 1280 respectively (Fig 4A), while heterologous GMT against D/660 virus were 80, 80, and 89 respectively. Similarly, all 3 ferrets inoculated with D/OK^660HEF^ variant seroconverted at 14 dpi. The mean homologous HI titers were 320, 452, and 452 respectively (Fig 4A), while the mean heterologous titers against D/OK virus were 160, 179, and 179 respectively (Fig 4A).

In the swine D/OK contact transmission group, consistent with viral titration data, 2/3 ferrets (T613 and T615) showing active viral replication (Fig 2C) seroconverted at 13 dpt (Fig 4B). The geometric mean homologous HI titers were 56, and 452 respectively (Fig 4B), while mean heterologous titers against D/660 virus were 10 and 56 (Fig 4B) respectively. In D/OK^660HEF^ variant contact transmission ferrets, 1/3 ferrets seroconverted at 13 dpt (Fig 4B). Notably, T610 ferret which showed detectable viral replication (Fig 2D) was the only animal seropositive by HI in the D/OK^660HEF^ variant transmission group. The geometric mean homologous HI titer was 80 (Fig 4B), while geometric mean heterologous titers against D/OK virus was 28 (Fig 4B). Collectively, our data indicated a strong association between infection/transmission and seroconversion, as animals with active virus replication were the only animals seropositive by HI. Statistical analysis using t-test was applied on log_2_ transformed HI titers in intranasally inoculated ferrets that were euthanized at 14 dpi. A statistically significant difference was observed in both homologous (*p* = 0.024) and heterologous (*p* = 0.0001) HI titers between D/OK and D/OK^660HEF^. These data also further support an early notion of the different antigenicity between swine D/OK and bovine D660 that is most likely driven by viral HEF protein.

### Detection of viral RNA (vRNA) in respiratory tissues of infected ferrets by RNAscope *in-situ* hybridization

**Fig 5:**
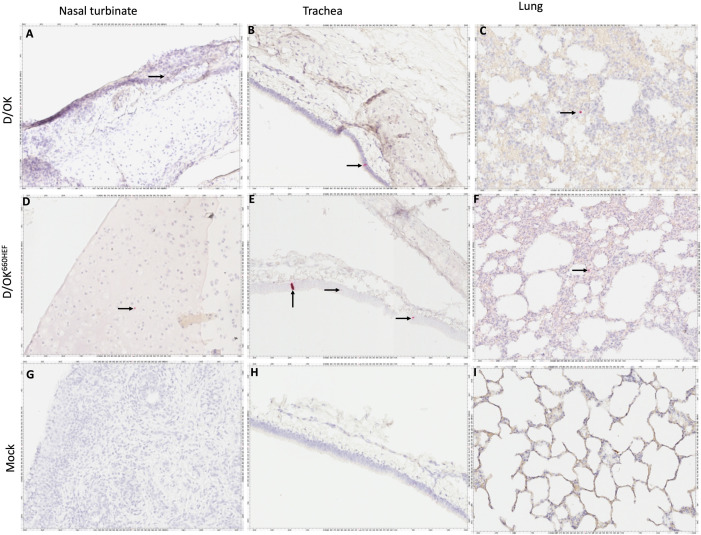
Detection of IDV viral RNA in ferret tissues at 4 dpi using RNAscope based *in-situ* hybridization (reddish pink, arrows). IDV PB1-specific vRNAs (reddish pink, indicated by arrows) were detected in D/OK inoculated ferret (T619) nasal turbinate (A), trachea (B), and lungs (C). Similarly, the D/OK^660HEF^ inoculated ferret nasal turbinate (T633) (D), trachea (T633) (E), and lung (T632) (F) also showed the presence of vRNA signals. Mock inoculated ferret (T606) nasal turbinate (G), trachea (H), and lungs (I) demonstrated absence of any signal. Images shown are representative of each of two groups.

**Fig 6:**
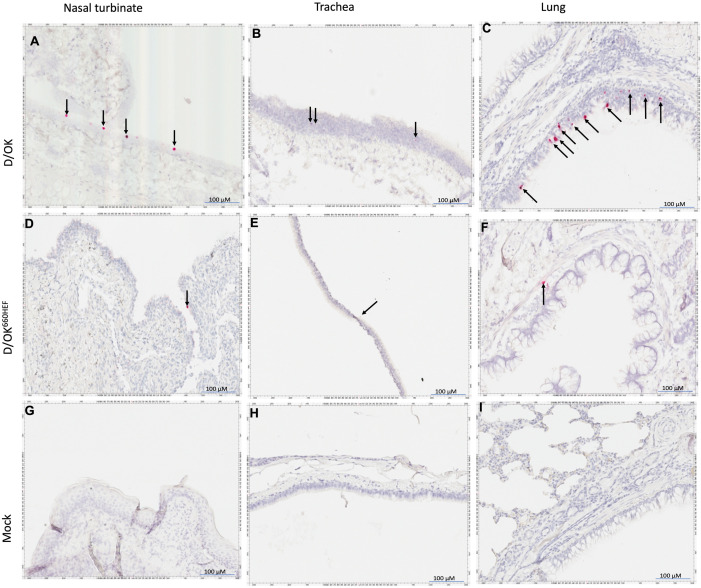
Detection of IDV viral RNA in ferret tissues at 14 dpi using RNAscope based *in-situ* hybridization. IDV PB1-specific vRNAs (reddish pink, indicated by arrows) were detected in D/OK inoculated ferret (T616) nasal turbinate (A), trachea (B), and lungs (C). Similarly, the D/OK^660HEF^ inoculated ferret (T628) nasal turbinate (D), trachea (E), and lung (F) also showed the presence of vRNA. Mock inoculated ferret (T608) stayed clean throughout the experiment as nasal turbinate (G), trachea (H), and lungs (I) demonstrated absence of IDV specific vRNA.

To further validate the tissue tropism observed with the cell-based IDV replication assay, we conducted RNAscope-based *in-situ* hybridization (ISH) to locate the vRNA genome in different tissues of the upper (URT) and lower (LRT) respiratory tract of infected ferrets, using negative-sense RNA probe derived from viral polymerase basic protein 1 segment (PB1) of IDV. As shown in Fig 5, the ISH experiment demonstrated the presence of IDV vRNAs in nasal turbinate tissues of infected ferrets with swine D/OK and D/OK^660HEF^ variant at 4 dpi (Fig 5A and 5D) and at 14 dpi (Fig 6A and 6D), respectively. IDV vRNAs were also detected in the soft palate tissues of these infected ferrets. In contrast, IDV vRNAs were not found in nasal turbinate, trachea, and lung tissues of mock infected ferrets at 4 dpi (Fig 5G - 5I) and 14 dpi (Fig 6G - 6I). Unexpectedly, *in-situ* hybridization detected IDV PB1 specific vRNA genome in lung and trachea tissues from swine D/OK and D/OK^660HEF^ variant inoculated ferrets at 4 dpi (Fig 5B - 5C and 5F). The positive signals were also present in these tissues till 14 dpi (Fig 6B - 6C and 6F). Despite infectious D/OK^660HEF^ particles detected in trachea (Fig 3F), viable swine D/OK particles were not found in this LRT compartment of infected ferrets (Fig 3C). Similarly, neither infectious swine D/OK nor D/OK^660HEF^ particles were detected in lungs of infected ferrets when samples were analyzed with the cell-based viral replication assay (Fig 3). Taken together, in addition to confirm the tissue tropism of swine D/OK and D/OK^660HEF^ variant as observed in the viral replication assay, ISH experiments further showed that both viruses may go through the entry process and replicate viral genome in ferret lungs, which could not be detected by the traditional virus isolation approach with infectious virus particles as the readout.

### Identification of IDV vRNA-positive lung and intestinal cells in infected ferrets

**Fig 7:**
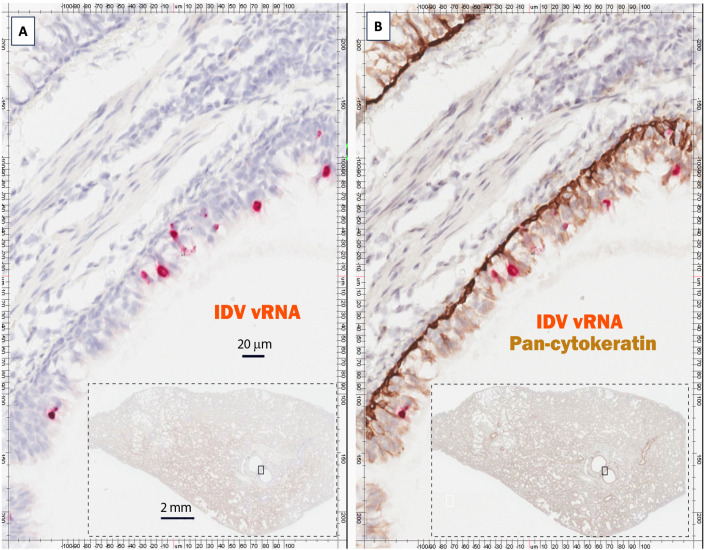
IDV vRNA signals are mainly localized in lung epithelial cells. To confirm vRNA were colocalized with lung epithelial cells, a section of lung tissue positive for vRNA in RNAscope was digitized (A), and subsequently immunohistochemically co-stained with pan cytokeratin antibody (B), indicating that majority of vRNA are localized with epithelial cells. The Figure A and B are highlighted respectively from a box of inset.

In order to annotate the specific cell types in ferret lung tissue where IDV vRNAs were detected, the lung cells following RNAscope ISH slides (Fig 7A) were co-stained with epithelial cell markers (Fig 7B). The viral RNA-positive cells were predominantly located within the epithelial cells, which have been identified using a pan-cytokeratin antibody (Fig 7B). To determine the cell type of few vRNA positive cells within interstitial tissues of the lungs, we co-stained RNAscope ISH with rabbit CD3 monoclonal antibody (Catalog number: RM-9107-S0, Thermo scientific, Kalamazoo, MI, USA), rabbit CD4 monoclonal antibody (Catalog number: ab133616, Abcam Waltham, MA, USA), mouse CD68 monoclonal antibody (Catalog number: ab955, Abcam Waltham, MA, USA) and rabbit CD31 monoclonal antibody (Catalog number: M3380, Spring Bioscience, Pleasanton, CA USA). Unfortunately, these antibodies did not cross-react with ferrets. As such, the precise cell types in ferret interstitial tissues of the lungs that were positive with IDV vRNA could not be determined here. Nevertheless, the results of *in-situ* hybridization coupled with IHC experiments indicate that the epithelial cells within the ferret respiratory tract are the major *in vivo* target cells of IDV infection where viral RNAs were evidently detected. Occasionally, viral RNA-positive cells were also observed within the interstitial tissues of the lung (Fig 7A and 7B). These positive cells are clearly not epithelial cells; however, the nature of these cell types could not be identified due to the lack of cross-reactive antibodies.

**Fig 8:**
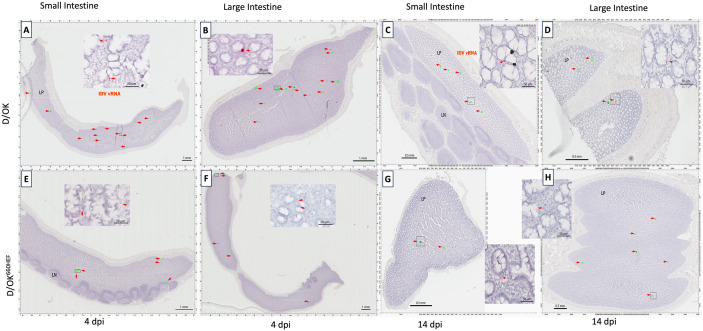
Detection of IDV vRNA in intestinal tissues using RNAscope based ISH. In D/OK infected ferrets, IDV specific vRNA (reddish pink, indicated by arrows) were detected in both small (A) and large intestine tissues (B) at 4 dpi (ferret T619) as well as in small (C) and large intestine (D) tissues at 14 dpi (ferret T616), where insets are magnified from small boxes of the whole tissue sections. Similarly, in D/OK^660HEF^ infected ferrets, IDV specific vRNA (reddish pink, indicated by arrows) were detected in both small (E) and large intestine (F) tissues at 4 dpi (ferret T632) and in small (G) and large intestine (H) tissues at 14 dpi (ferret T628).

Similar to lung tissues, no viable viruses were also recovered from fecal swabs and intestinal tissues of infected ferrets. However, RNAscope-based ISH demonstrated the presence of IDV vRNAs in the small and large intestine of ferrets intranasally inoculated with swine D/OK and euthanized at 4 dpi (Fig 8A - 8B) or at 14dpi (Fig 8C - 8D). Similarly, IDV PB1 specific vRNAs were detected in the small and large intestine of ferrets intranasally inoculated with D/OK^660HEF^ variant and euthanized at 4 dpi (Fig 8E - 8F) or at 14 dpi (Fig 8G - 8H). While the intestinal tissue was not co-stained with pan-cytokeratin antibodies, the whole slide imaging of RNAscope ISH data indicated that vRNA positive cells were localized in the lamina propria (LP) of the gut tissues but not in the gut-associated lymph node tissues (LN) (Fig 8A - 8H). In the gut, numerous vRNA positive cells were identified as endothelial/epithelial cells based on location and morphology, with some positive cells also found in the LP that were not epithelial or endothelial cells. Collectively, results of RNAscope-based ISH analysis suggest that IDV can enter ferret intestinal cells with a successful replication of its genome in the gut. This hypothesis appears to be supported by published work showing that IDV can replicate in the intestine of experimentally infected mice or shed in feces of naturally infected small ruminant [53,54]. Future study is needed to evaluate the potential fecal-oral transmission of IDV in animals in addition to identify the specific cell type in the intestine that can replicate IDV.

### Association between vRNA expression and viral load in ferret tissues and quantitative analysis of IDV receptor in ferret lung

**Fig 9:**
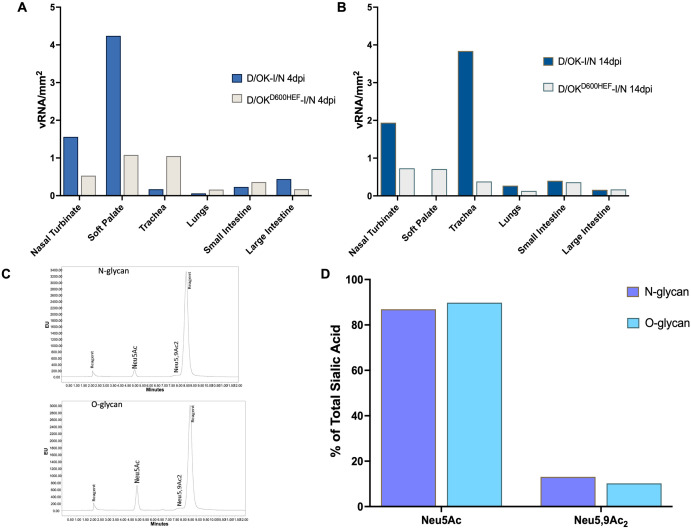
Quantification of vRNA in various tissues and sialic acid profiling of ferret lung tissue. IDV specific vRNA were quantified and expressed as vRNA/mm^2^ of tissue area. Viral RNA quantification in respiratory and intestinal tissues from intranasal inoculated ferrets euthanized either at 4 dpi (A) or 14 dpi (B). Ferret lung tissue was subjected to sialic acid profiling to confirm presence of IDV specific receptor. (C) Ferret lung tissue exclusively synthesizes Neu5Ac and indicated presence of its 9-*O*-acetylated derivative; Neu5,9Ac_2_. (D) Quantification of sialic acid indicated presence of sialic acid associated with N- and O-linked glycans, and Neu5Ac being the predominant form of sialic acid. vRNA signals were quantified for images from ferret tissue shown above in Figs 5–6 and 8.

To determine whether there is an association between vRNA expression level detected by ISH and viral load measured by the cell-based replication (Fig 3), we performed a quantitative analysis of ISH images by manual quantification of vRNA-positive cells from digitized image of the stained tissue sections and determined the level of vRNA expression as a function of each mm^2^ of tissue section. Ferret T619 and Ferret T616 from swine D/OK group were selected as representatives of 4 dpi and 14 dpi subgroups, while Ferret T633 and Ferret T628 from D/OK^660HEF^ variant group were used as representatives of 4 dpi and 14 dpi subgroups in this analysis. As shown in Fig 9A and 9B, swine D/OK infected ferret expressed higher levels of vRNA in the upper respiratory tract (nasal turbinate and soft palate) than those in the lower respiratory tract (trachea and lung) and the intestinal tract at 4 dpi. This tissue-specific vRNA expression abundance correlated well with the virus replication data obtained from 4 dpi in that viral titers were measurable only in nasal turbinate and soft palate, not in other tissues (Fig 3A-3C). A similar trend was also observed in swine D/OK infected Ferret T616 at 14 dpi. Interestingly, trachea in this ferret at 14 dpi expressed a significant amount of vRNA (Fig 9B) but derived no viable viruses (Fig 3C). For D/OK^660HEF^ variant group, both 4 dpi (T633) and 14 dpi (T628) ferrets exhibited a similar trend in terms of vRNA expression levels measured in various tissues (Fig 9A-9B). For Ferret T633 at 4 dpi, substantial amounts of vRNA expression were found in the respiratory tract tissues except for lung, while relatively lower levels of vRNA were detected in the small and large intestinal tissues (Fig 9A). vRNA expression levels in nasal turbinate, soft palate, and trachea agrees well with viral isolation and titration data on these tissues (Fig 3D-3F).

By comparing vRNA levels in nasal turbinate, soft palate, and trachea between swine D/OK and D/OK^660HEF^ infected ferrets, it appears that swine D/OK infected ferrets expressed relatively higher levels of vRNA in these respiratory issues than D/OK^660HEF^ infected animal. Intriguingly, this vRNA data do not directly translate viral titers measured in the viral replication experiment because viral titers derived from both viruses in ferrets were similar (Fig 3). Future experiment is needed to address this discordance. Similar questions concerning the biological implications of viral genome present in the lung and intestinal tract of ferret without viable viruses recovered from these tissues should be studied as well.

To further elucidate the potential of IDV to enter and replicate in ferret lung cells, we conducted a high-performance liquid chromatography (HPLC) analysis to determine whether IDV receptor is present in the lung cells of ferret. Previous studies have shown that IDV binds to sialosides terminated with a Neu5,9Ac_2_ or a Neu5Gc9Ac and use them as functional entry receptors for infection [31]. As shown in Fig 9C - 9D, HPLC analyses revealed that ferret lung tissue expressed Neu5,9Ac_2_ (IDV receptor) in addition to a high amount of non-modified Neu5Ac. Neu5,9Ac_2_ was detected in both N-glycan and O-glycan forms (Fig 9C - 9D). This data supports the ability of IDV in entering and replicating its genome in lung cells, further strengthening the finding of IDV-specific vRNAs detected in lung tissues of ferrets (Figs 5 - 7 and 9).

### High-throughput sequencing of viruses isolated from tissue samples of ferrets infected with swine D/OK and D/OK^660HEF^ variant

The sequences from clinical materials had low read coverage and read depth and therefore, we opted for sequencing of P0 supernatants obtained right after inoculation of MDCK cells. The swine D/OK inoculum sequence had only one amino acid difference (I4N) in PB1 segment when compared to the reference sequence. This PB1 I4N substitution in the viral inoculum was also present in all the clinical ferret samples from the swine D/OK. Interestingly, we found D87Y mutation in PB2 in nasal turbinate of swine D/OK inoculated ferrets that were euthanized at 4 dpi. In swine D/OK group, E734K in PB1 and I321T in P42 were two additional mutations identified in nasal washes at 6 dpi and 7 dpt, respectively. Intriguingly, these two mutations were not identified in ferrets infected with D/OK^660HEF^ variant, despite two viruses possessing identical PB1 and P42 segments. In D/OK^660HEF^ variant inoculated ferret, we observed one substitution (A27V) in PB1 in soft palate, which was not present in soft palate of swine D/OK infected ferrets. This soft palate sample also showed presence of synonymous variant in P3 (T1371C) resulting in no amino acid change (F457F) (Table 1). Neither swine D/OK HEF nor bovine D/660 HEF showed notable amino acid sequence changes, indicating no adaptation mutation needed for swine and bovine HEF in driving IDV to efficiently replicate and transmit in ferrets.

**Table 1:** Single nucleotide polymorphisms observed in clinical samples of infected ferrets.

Sample/Ferret group	SNP Frequency (%)	Mutation	Gene
T615_7 dpt_Nasal Wash (D/OK_Contact Transmission group)	99.90	Nucleotide: T962C Protein: Ile321Thr	P42 (DM2 protein)
T617_6 dpi_Nasal Wash (D/OK_ I/N inoculated group)	93.43	Nucleotide: G2200A Protein: Glu734Lys	PB1
T619_Nasal Turbinate_4 dpi (D/OK_I/N inoculated group)	99.06	Nucleotide: G259T Protein: Asp87Tyr	PB2
T632_ Soft palate_4 dpi (D/OK^660HEF^ I/N inoculated group)	95.31	Nucleotide: C80T Protein: Ala27Val	PB1
	91.70	Nucleotide: T1371C Protein: Phe457Phe	P3

*(**Ferret group/sample**: ferret group and ferret ID for which these mutations were observed. **SNP Frequency**: % of sequencing read frequency counts for alternate nucleotide. **Mutation**: The effect of SNP (number indicates the location corresponding to the coding sequence). **Gene**: The gene or protein of IDV.

### Pathological changes in respiratory tract tissues of infected ferrets

**Fig 10:**
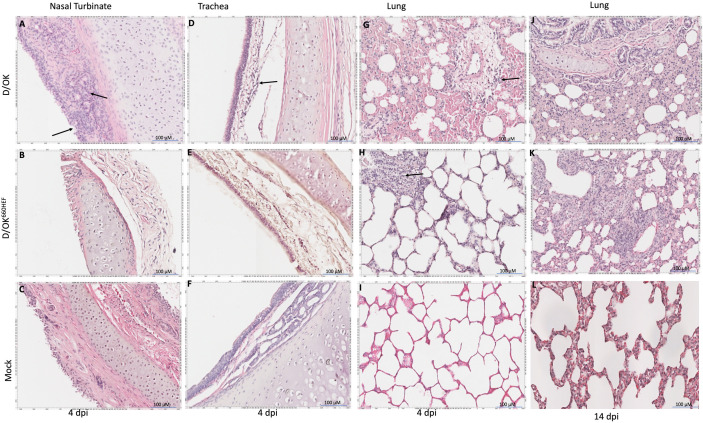
Histopathological changes in the nasal turbinate, trachea, and lungs upon inoculation with D/OK and D/OK^660HEF^ viruses in ferrets. D/OK inoculated ferret nasal turbinate (A), trachea (D), and lungs (G) showed mild to moderate inflammation at 4 dpi, which persisted until 14 dpi for lung tissue (J). D/OK^660HEF^ inoculated ferret nasal turbinate (B) showed loss of epithelial lining while trachea (E), and lungs (H) showed mild to moderate inflammation at 4 dpi, which persisted until 14 dpi for lung tissue (K). No inflammatory changes were observed in mock inoculated ferret nasal turbinate (C), trachea (F), and lung (I) at 4 dpi. Slight thickening of alveolar septa of mock lung at 14 dpi (L) was observed compared to 4 dpi, no inflammatory changes were observed. Representative images used for histopathological analysis were derived from the same ferrets used for vRNA detection in Figs 5-6.

IDV infected ferrets did not show any appreciable gross lesions in the respiratory tract tissues. We conducted the H&E staining of respiratory tissues for determining any histopathological changes in ferrets. Intranasal inoculation of swine D/OK and D/OK^660HEF^ variant resulted in mild to moderate pathological changes in the respiratory tract tissues of ferrets at 4 dpi (Fig 10). At 4 dpi, swine D/OK infected ferrets showed the notable inflammation of the nasal epithelia, and disruption of epithelial lining with moderate infiltration of inflammatory cells, especially lymphocytes and macrophages in the lamina propria and submucosa of nasal turbinate tissue (Fig 10A). Furthermore, the epithelial lining was missing in nasal turbinate derived from D/OK^660HEF^ invariant infected ferrets (Fig 10B), however it could not be ascertained if this was result of pathological change or artifact introduced during tissue sectioning. In trachea, both swine D/OK and D/OK^660HEF^ variant infection did not result in disruption of epithelial lining. Nevertheless, moderate infiltration of inflammatory cells was observed (Fig 10D and 10E). Pathological changes were also observed in the lungs of ferrets infected with both viruses at 4 dpi (Fig 10G and 10H). The swine D/OK infected ferret lung showed the presence of inflammation and exudate with diffused infiltration of inflammatory cells, mild perivascular cuffing, and thickening of alveolar epithelium (Fig 10G). The pathological changes appeared to be mild for D/OK^660HEF^ variant inoculated ferret lungs. Nevertheless, a few focal areas of inflammatory cell infiltrations were observed (Fig 10H). In contrast, all three tissues (nasal turbinate, trachea, lungs) from mock inoculated ferrets showed no pathological changes at 4 dpi (Fig 10C, 10F, 10I, and 10L). While we observed mild thickening of alveolar septa at 14 dpi in mock inoculated ferret lungs (Fig 10L), the alveolar spaces seemed to be normal. Notably, the inflammatory changes along with narrowing of intra-alveolar spaces due to inflammatory infiltrate have persisted in lungs until 14 dpi in both swine D/OK or D/OK^660HEF^ variant infected ferrets (Fig 10J and 10K).

### Structure modelling of swine D/OK and bovine D/660 HEF proteins

**Fig 11:**
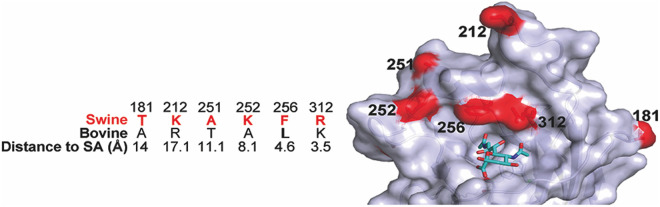
Structure modelling of swine D/OK and bovine D/660 HEF proteins. The modelled bovine D/660 HEF structure is superimposed on the HEF structure of swine D/OK HEF (PDB: 5E64). Six mutations between bovine D/660 and swine D/OK distal to the receptor binding pocket (RBP) of the HEF protein are indicated and their respective distance to the RBP are described. A Neu5,9Ac_2_ receptor analog is shown in green in the context of the HEF structure.

Our previous study on glycan binding differences between swine D/OK and bovine D/660 has shown that there are subtle differences in receptor binding specificities between these two viruses [55]. Further analysis and comparison of the viral HEF protein structure revealed that both viruses possessed the identical receptor binding pocket (RBP) but were variable in six surface-exposed positions distant from the RBP (3.5 to 17.1 angstrom) (Fig 11). HEF position 212 has been previously implicated in imparting an approximate 10-fold antigenic difference to these two IDV clades [47]. Impact of these HEF polymorphisms on receptor specificity, tropism, and transmission efficiency between swine D/OK and bovine D/660 should be investigated in future study.

## Discussion

To date, more than 5 different HEF based phylogenetic clades, and 3 additional subclades are reported, and of these diverse IDV groups, D/OK and D/660 clades are dominant in circulation among the global cattle population [56]. These two phylogenetic clades have maintained sustained transmission in US, Europe, and Japan, in addition to recent identification in Africa and China [57,58]. Their high transmissibility, immune evasion ability, and worldwide appearance have raised global concerns for cattle industry. Furthermore, IDV genomes, in addition to antibodies, were also detected in humans in multiple studies [1,24–27], indicating its potential in infecting and causing influenza-like illness in humans. As such, it is imperative to conduct a comprehensive study in ferrets, a surrogate model of human influenza, to characterize IDV in terms of replication fitness, transmission efficiency, and pathogenesis.

Multiple lines of evidence support that IDV HEF protein binds to both 9-*O*-acetyl-*N*-acetylneuraminic acid (Neu5,9Ac_2_) and non-human 9-*O*-acetyl-*N*-glycolylneuraminic acid (Neu5Gc9Ac) and utilizes them as functional receptors for viral entry [31,51,55]. These studies also indicate that IDV HEF protein is a critical determinant of its broad tropism and sustained transmission among different animal species. This study aimed to take advantage of the ferret model to examine and compare the contribution of different HEF proteins to viral replication fitness, tissue tropism, and transmission efficiency. We selected swine D/OK and bovine D/660 HEF proteins representative of two dominant IDV clades (D/OK and D/6600) for this work. Specifically, we leveraged a recently developed swine D/OK RGS [52] and utilized it to generate an isogenic swine D/OK virus expressing bovine 660 HEF protein. These two viruses (swine D/OK and D/OK^660HEF^) that vary only in HEF protein were fully characterized in ferrets towards elucidating a role of HEF protein in IDV replication fitness, tissue tropism, and transmission efficiency. While studying two isogenic viruses differing only in HEF protein in ferrets enabled us to observe the HEF-dependent tropism and transmission differences between two dominant IDV clades D/OK and D/660, it should be noted that these HEF-specific observations were made in the context of the D/OK only. Future studies aiming at directly comparing the wild-type D660 and its derivative expressing D/OK HEF protein are needed to gain a full picture of viral determinants including HEF protein for driving the divergent tissue tropism and transmission efficiency between two IDV clades in ferrets and other animals.

The infection dose and route used in this ferret experiment are routinely employed for experimental infection of ferrets with influenza A viruses [59,60], including the first and the only ferret study with IDV [1]. Intranasal inoculation of both swine D/OK and its D/OK^660HEF^ variant did not result in any detectable clinical diseases in ferrets despite active viral replication detected in nasal turbinate, soft palate, and trachea. This subclinical nature of disease was also reported in guinea pigs and mice experimentally infected with IDVs [1,61]. Ferrets inoculated with swine D/OK virus showed viral replication mostly in nasal turbinate, and soft palate, while no replication was observed in trachea or lung. In contrast, D/OK^660HEF^ variant infected ferrets demonstrated that in addition to the URT compartments (nasal turbinate and soft palate), viral replication was also detected in trachea. Two ferrets (T620 and T625) necropsied on 4 dpi did not yield detectable infectious virus particles from the nasal turbinate tissue as measured in the standard cell-based viral infectivity (TCID50) assay. To further investigate this issue, we performed IDV-specific RT-qPCR assay on respiratory tract tissues (nasal turbinate, soft palate, trachea, and lungs) using previously described protocol [12]. As shown in S1 Fig, trachea samples from both ferrets (T620 and T625) tested weakly positive by RT-qPCR, with Ct values of 32.5 and 34.4 respectively. Based on the standard curve (Ct vs viral titer), these corresponded to estimated viral titers of 10^2.17^ TCID_50_/ml and 10^1.61^ TCID_50_/ml respectively; at or below the detection limit of our cell based TCID_50_ assay. In addition, we also found soft palate sample from T620 ferret and nasal turbinate sample from T625 ferret were tested weakly positive with Ct 37.09 and Ct 36.15, respectively. This result indicates that both ferrets were infected, undergoing some levels of viral replication in the respiratory tract of infected ferrets. Although both animals were inoculated with the same infectious dose as other ferrets shedding significant amounts of viruses, unavailability of nasal wash samples from these two ferrets limits our ability to further interrogate the viral shedding kinetics and infection nature of IDV in these two animals. It is possible that productive infection was not established in these two ferrets, and its viral yield is below the detection threshold of the cell-based virus replication assay used in this study. This highlights the importance of collecting multiple specimen types and using various time points to characterize infection dynamics of IDV in ferrets. Furthermore, the different levels of IDV replication in ferrets may indicate a role of host genetic variability and the associated innate immune response, which should be addressed in future work. Despite these limitations, the results of our experiment suggest that bovine D/660 HEF may contribute to its ability to drive IDV to replicate in the low respiratory tract of ferret.

It should be noted that some discordances between nasal wash titer and tissue titer were observed in two ferrets. Ferret T616 showed early virus clearance in nasal washes but exhibited a higher number of infectious virus particles in nasal turbinate collected following the terminal euthanasia. Interestingly, another ferret T626 demonstrated prolonged shedding of IDV in nasal wash but failed to show detectable infectious virus particles in nasal turbinate tissue. This phenomenon (persistent viruses detected in tissues without shedding in the nasal washes) is also reported in ferrets infected with seasonal influenza A viruses, where infectious viruses remained detectable in tissues after they were no longer recoverable from nasal washes [62,63]. In our study, detection of IDV in nasal turbinate tissue at 14 dpi in the absence of concurrent shedding in nasal washes indicates a possibility that IDV may persist longer in the upper respiratory tissues.

Conversely, failure to detect infectious virus particles in nasal turbinate of a ferret despite low-level shedding in nasal washes could reflect the localized host-specific immune clearance or sampling limitations. Moreover, this study did not analyze host immune response to IDV, which is a critical factor in regulation and clearance of viral infection. Group size of ferrets used in this study is another limitation. Thus, these findings warrant further investigation towards better understanding of IDV tissue tropism and the dynamics of viral clearance across anatomical sites as well as anti-IDV host immune response and its effect on viral persistence at different anatomical sites.

Viral shedding through the nasal secretions is critical to successful transmission of the virus to contact naïve ferrets. Seroconversion is additional measurement that can be used to determine viral transmission from infected ferrets to sentinels [62]. Viral shedding data combined with the results of tissue tropism and seroconversion experiments obtained in this study collectively suggest that swine D/OK virus exhibited slightly better transmission efficiency than D/OK^660HEF^ variant in ferrets. Although bovine D/660 HEF may play a role in facilitating viral replication in the lower respiratory tract (LRT), swine D/OK HEF appears to potentially provide a relative advantage in promoting viral transmission efficiency in ferrets. Interestingly, studies on IAV transmission using ferrets have shown that the upper respiratory tract is the anatomical site for generation of airborne IAV, and that viruses replicating in the URT (nasal turbinate) preferentially transmit to naïve ferret when compared to those present in the LRT [64].

Among at least five phylogenetic clades of IDVs, D/OK and D/660 are two dominant groups that are circulating in global cattle population [46,56]. Previous studies have shown that HEF is the primary determinant of acid and thermal stability observed in IDV, and that the HEF proteins from D/OK and D/660 clades differ in the tissue microarray-based binding profile, protein histochemical staining, and lectin binding experiment [51,65,66]. Since the two viruses used in our study were isogenic except for HEF, the phenotypic differences in tissue tropism and transmission efficiency can be attributed to functional differences in HEF proteins of swine D/OK and bovine D/660. The primary amino acid alignment reveals that swine D/OK HEF protein varies from bovine D/660 HEF by 20 amino acid sequences. The structural analysis further shows that among these polymorphisms, six amino acid changes between swine and bovine HEF proteins occur in the receptor binding domain, which are represented by T181K, K212R, A251T, K225A, F256L, and R312K (listed are the swine D/OK amino acid residue, position, and bovine D/660 amino acid). These six amino acid differences in the receptor binding domain may have a pivotal role in dictating replication fitness, tissue tropism, and transmission efficiency of swine D/OK and bovine D/660 viruses in ferrets. It is further evident from our previous glycan microarray experiment that two HEF proteins possess different receptor fine specificities, despite exhibiting overlapping binding specificities [55]. Intriguingly, bovine D/660 HEF has a broader receptor specificity, and its glycan-binding appears to be not affected substantially by sialyl linkages (α2–3/6) and the complex of glycans bearing a terminal 9-*O*-acetylated sialic acid. In contrast, swine D/OK HEF possesses a narrower receptor specificity with the preference to sialosides with specific linkages and internal glycans. Thus, it can be inferred that HEF polymorphisms, especially associated with the receptor binding domain may be the primary determinants of the observed difference in tissue tropism and transmission efficiency in ferrets infected with swine D/OK and its variant expressing bovine D/660 HEF. Future study is needed to address how HEF‐specific mutations affect tissue tropism and influence the transmission efficiency between swine D/OK and bovine D/660 viruses in ferrets.

As with influenza A viruses, previous studies have demonstrated both host- and virus strain-specific tissue tropism. Experimental inoculation of pigs with IDV strains from D/OK clade resulted in no clinical disease, with active viral replication primarily confined to the URT in domestic pigs [1,53,67]. A separate study, involving experimental challenge of pigs with a IDV strain from D/660 clade however showed that, IDV could replicate in lungs with visible lung lesions [68]. Similarly, calves infected with IDV from D/660 clade developed moderate respiratory disease, with viral RNA found in both the upper and lower respiratory tracts [69]. In guinea pigs, D/660 clade virus replicated in both URT and LRT including lungs [61]. Small animal models are thought to provide more tractable infection studies than large animal models. So far, guinea pigs, mice, and a single ferret study [1,54,61] have been reported for IDV transmission and pathogenesis study. A mouse model of IDV infection with an IDV strain from D/OK clade indicated viral replication mainly in the URT [54], while guinea pig studies with D/OK virus showed robust viral replication in both URT and LRT including lung [43,61]. The only ferret study with swine D/OK showed viral replication only in nasal turbinate [1]. Thus, experimental observations in our ferret study were congruent with previous work conducted in experimentally infected large animals (pigs and bovines) and small animals (mice, guinea pigs, and ferrets) with IDVs. Nevertheless, the current work further extends a previous ferret infection experiment in that it shows a HEF-dependent tissue tropism and transmission efficiency, especially manifested by swine D/OK and bovine D/660 HEF proteins, which should provide the foundation for further mechanistic investigation of the HEF-mediated replication and transmission mechanisms in ferrets and other animal species.

Although no infectious viruses were recovered from lungs of infected ferrets, vRNAs were detected in lungs of infected ferrets with both swine D/OK and its mutant expressing bovine D/660 HEF. In contrast, previous guinea pig model of IDV infection showed robustly viral replication in guinea pig lungs [61]. Although, sialic acid species in ferrets and guinea pig are different, with guinea pigs expressing both Neu5Ac, Neu5Gc, and their 9-*O*-acetylated forms (Neu5,9Ac_2_ and Neu5Gc9Ac), and ferret only expressing Neu5Ac and its 9-*O*-acetylated form Neu5,9Ac_2_ [38,43,70], IDV can utilize either Neu5,9Ac_2_ or Neu5Gc9Ac as a functional receptor for entry and infection [31]. Based on the UHPLC-based analysis in this study showing the presence of IDV receptor Neu5,9Ac_2_ coupled with the detection of vRNA in ferret lung tissue, we reasoned that ferret lung cells are permissive to IDV entry and its genome replication. This notion is also supported by the presence of pathological changes in lungs of infected ferrets at both 4 and 14 dpi in which vRNAs were detected. It should be noted that the PB1 probe used in RNAscope-based ISH experiment could not distinguish the full-length vRNA segment from those derived from defective forms containing the central deletions in the PB1 segment. Regardless of the vRNA source, the presence of vRNA signals provides evidence that IDV successfully enters and replicates its genome in ferret lung cells. Two hypothetical models could explain the presence of measurable vRNAs, in the absence of infectious virus particles detected in ferret lungs. The first model is that IDV could not establish a robust or sustained infection in lung epithelial cells. Since viral genome replication was detected in lung cells expressing IDV’s functional Neu5,9Ac_2_ receptor, “abortive replication” would occur in the late stage of viral replication such as assembly and release. The other model is that IDV could not effectively overcome intrinsic restriction factors or local interferon-inducible antiviral effectors residing in ferret lung cells so active viral replication would be substantially inhibited. According to this model, inhibition of IDV replication would be specific to ferret lung cells as IDV replication is evidently detected in lung cells of other animal species such as guinea pigs and bovines as well as the epithelial cells lining the upper respiratory tract of ferret.

The proposed two models may also explain the observations of IDV infection in the intestinal tissues of ferrets where vRNA signals occurred and persisted in the gut tissues primarily in the intestinal epithelium which derived no viable virus particles. Detection and persistence of vRNAs in the small and large intestine may indicate a fecal-oral transmission as a minor mode of spread for IDV. A previous mice study showed IDV replication in the mouse intestine after experimental inoculation of IDV [54]. IDV shedding was also detected in goat fecal materials [53]. Moreover, the intestines express higher levels of *O*-acetylated sialic acids, including 9-*O*-acetylated sialic acids, which serve as a functional receptor for IDV [30]. In birds, fecal-oral transmission is the predominant route of transmission although virus is shed in nasal secretions as well [71]. Viral RNAs of several respiratory viruses including SARS-CoV, MERS-CoV, and influenza A virus are frequently detected in feces of humans [72], however, the significance of transmission in causing clinical disease is unknown. Thus, the potential of fecal-oral transmission of IDV and its significance in disease spread in native animals such as cattle and swine need further investigation especially considering growing evidence of fecal-oral transmission in respiratory enveloped RNA viruses.

Deep RNA sequencing of viruses derived from the inoculum and post-inoculation samples revealed no amino acid changes in HEF proteins of swine D/OK and bovine D/660. This finding suggests that no adaptation mutation is needed for the HEF protein to efficiently replicate and transmit in ferrets. Nevertheless, mutations were identified in the other segments of viruses derived from infected ferrets when compared with the genome sequence in the viral inoculum. The observed mutations included PB1 E734K, PB1 A27V, PB2 D87Y, and P42 (DM2) I321T. Future experiments are needed to characterize if observed mutations have any adaptive advantage in viral replication and transmission, or if these are just random mutations. While experimental data on these mutations in IDV is not available, it can be inferred based on the roles of the polymerase complex and M2 proteins in influenza A viruses, which are heavily investigated over the past several decades. IDV PB1 E734K mutation is close to the C-terminus of the catalytic subunit of RNA-dependent RNA polymerase complex (RdRP). Previous studies on influenza A viruses show that E627K mutation in the PB2 segment of avian influenza H7N9, and H5N1 is associated with mammalian adaptation [73,74]. While the role of PB1 E627K mutation observed in ferrets is unknown in IDV replication fitness and host range, the substitution of negatively charged to positively charged residue at position 627 in PB1 which is the catalytic subunit of RdRP may have profound implications in virus adaptability and replication fitness [75]. The P42/M segment splicing strategy in IDV is unique, in that it adds additional 4 amino acids into preceding exon after splicing for generation of M2, which is different from its closely related ICV [1]. The observed mutation in P42/M segment is on DM2 protein. While the detailed structure-function of IDV DM2 is yet to be fully characterized, the structural functional studies of influenza A virus suggests that M2 protein is an ion channel protein with possible role in maintenance of appropriate pH across viral and trans golgi membranes, which is necessary for viral maturation and subsequent entry [76]. In addition, M2 protein of ICV has been shown to play a role in genome packaging [77]. The substitution of isoleucine (I), a non-polar hydrophobic amino acid, with threonine (T), a polar amino acid could be an adaptive strategy to improve the rate of proton flux [76]. Both AM2 and BM2 (IAV and IBV M2 protein respectively) have numerous polar residues and the substitution of these polar residues with more hydrophobic amino acid results in a lower rate of proton flux [78], thereby potentially affecting viral uncoating and viral genome release in cytoplasm. Thus, further investigation into structure of IDV DM2 and its role in IDV replication need to be explored. Interestingly, of all the publicly available IDV protein sequences at time of the analysis (136 PB2 sequences, 121 PB1 sequences, and 179 P42 sequences), none of the observed mutation were present in respective sites of these sequenced segments from bovine and swine IDVs. The amino acids occupying these positions in naturally occurring IDV strains are absolutely conserved, emphasizing that these native amino acid residues may play an essential role in IDV replication and transmission in cattle and swine. Thus, these observed mutations specific to ferrets could be key adaptive mutations that may facilitate replication fitness and transmission in ferrets, which warrant further investigation. Ferrets have been extensively used for pandemic or zoonotic risk assessment posed by animal influenza viruses. Successful replication and transmission of IDV in ferrets as shown in this work further support the utility of ferrets as an animal model to study this new type of influenza viruses and evaluate its potential to replicate in humans.

## Materials and methods

### Ethics statement

Animal experiment was conducted as per the approved protocol, guidance, and regulation of Institutional animal care and use committee (IACUC) at the University of Kentucky (IACUC approved protocol number 2020–3622).

### Cells and viruses

Madin-Darby canine kidney (MDCK) cells, and HEK-293T (Human embryonic kidney) cells were cultured in Dulbecco’s minimum essential medium (DMEM; Gibco, Invitrogen, USA) supplemented with 10% (v/v) fetal bovine serum (FBS; Gibco, USA) and 100 U/ml penicillin-streptomycin (Life Technologies, Carlsbad, CA, USA). IDV swine D/OK (D/swine/Oklahoma/1334/2011) [1] and bovine D/660 (D/bovine/Oklahoma/660/2013) [2] were originally isolated from swine and cattle, respectively, and these two isolates are prototype strains representing D/OK and D/660 clades. The detailed protocol for rescue of IDV swine D/OK virus using the reverse genetics system (RGS) has been described in our previous work [52]. For generation of D/OK^660HEF^, a plasmid encoding the HEF of D/bovine/Oklahoma/660/2013 replaced swine D/OK HEF plasmid, while the rest of the segments were from swine D/OK RGS. Following transfection, culture supernatants were propagated on MDCK cells. After inoculation of the supernatants for 1 hour, the inoculum was discarded, and cells were washed once with 1X PBS. The cells were then incubated at 37°C in 5% CO2 for 5 days in fresh DMEM supplemented with 1 μg/ml of TPCK-treated trypsin (Catalog number: 20233, ThermoFisher, Carlsbad, CA, USA). Following the incubation period, the resulting supernatants were centrifuged at 500 × g for 10 minutes at 4°C to eliminate cellular debris. The virus titers were determined using the Reed and Muench method [79]. Viral loads in the nasal washes and tissue homogenates were titrated on MDCK cells using Reed and Muench method as well.

### Animals

Three and half month old, female, influenza viruses-seronegative ferrets (*Mustela putorius furo*) were obtained from Triple F farm (Triple F Farms LLC, PA, USA). Ferrets were housed in ventilated negative-pressure rabbit cages (Allentown LLC). Purified air was supplied to the cage using HEPA filter. Ferrets body weight, at time of arrival, ranged from 510–613 grams. The ferrets were ear tagged for the identification purpose and were given two weeks acclimatization period. Ferret sera were screened for IDV specific antibody and were all negative for IDV. Total experiment duration was 4 weeks, that included 2 weeks of acclimatization post arrival. Ferrets were provided with food and water ad libitum and kept on a 12-hour light/dark cycle. Control animals were sampled before the inoculated animals and strict precautions were taken to prevent cross contamination, among groups. Sentinel animals were sampled before inoculated animals in transmission experiments.

### Experimental design

At day 0, eight ferrets were mock challenged with sterile 1X PBS (500 μl/nostril). Mock controls were respectively euthanized at day 0 (n = 4), day 4 (n = 2; control for replication kinetics study), and at day 14 (n = 2; control for transmission study). Similarly, 14 ferrets were respectively challenged with 3.98 x 10^5^ TCID_50_/ml of the virus, 500 μl/nostril of D/OK (n = 7), or D/OK^660HEF^ (n = 7). Four of the seven challenged ferrets (for both D/OK and D/OK^660HEF^) were euthanized at day 4 for replication kinetics and tissue tropism studies, while 3 ferrets from each group were paired with 3 sentinels thus making total 6 ferrets each in the transmission study groups. The ferrets in the transmission study groups were euthanized at 14-days post challenge. Fig 1 shows the schematic representation of the experimental design. During the infection and nasal wash collection, ferrets were sedated using dexmedetomidine hydrochloride (Dexdomitor 0.04 mg/kg IM) and were maintained under 2–3% isoflurane anesthesia. The sedative effect of Dexdomitor was reversed by I/M injection of Atipamezole (Antisedan) at required dosage (at the same volume as the Dexdomitor). For euthanasia, ferrets were first presedated with dexmedetomidine hydrochloride (Dexdomitor 0.04 mg/kg IM) and maintained under 3% isoflurane for deep anesthesia. This was followed by intravenous administration of saturated potassium chloride solution (through cranial vena cava).

### Clinical sign monitoring and sample collection

Prior to the virus challenge, body weight and temperature of ferrets were measured. Blood was collected from all of the animals through the cranial vena cava under the anesthesia conditions mentioned above. Animals were monitored daily after virus challenge for clinical signs, while body weight and temperature were recorded every other day. Auricular temperatures were recorded using tympanic digital thermometer. The nasal washes were collected by instilling 1 ml of PBS using 20 G x 1.5-inch oral gavage needles (ferrets were held at a downward slope to prevent any aspiration into the lungs) and were then allowed to sneeze, which was subsequently collected on a petri dish. Nasal washes collected in the petri dishes were transferred to 1.5-ml tubes and then centrifuged at 500 × g for 5 min at 4°C to remove any debris. Nasal washes were not collected from ferrets euthanized at 4 dpi. Fecal swabs were also collected from these ferrets and were processed by resuspending in 1ml of PBS, followed by vortexing, and centrifugation. Clear supernatants were then collected and stored at -80°C until further use. Post euthanasia, blood, nasal turbinate, soft palate, trachea, lungs, and intestines were collected for transmission and tissue tropism studies. After collection, all clinical samples were stored at -80°C until further use.

### Viral replication kinetics and tissue tropism

For the preparation of tissue homogenates, clinical samples including nasal turbinate, and soft palate tissue were resuspended in 1ml of DMEM supplemented with penicillin-streptomycin (200 U/ml) and homogenized using a bead beater homogenizer (2500 of two pulses, 30 second each for a pulse). For lung, trachea, and intestine, 1 gram of tissue was resuspended in 1ml of media followed by homogenization. The resultant homogenized tissue fluids were then clarified by centrifugation at 500 × g for 10 minutes at 4^o^C and processed for virus titration. To determine the virus titer in the nasal washes and tissue homogenates, MDCK cells were seeded in 96-well tissue culture plates (20,000/well) and allowed to grow overnight. Next day, the cell culture plates were washed with sterile 1X PBS. The viral samples were serially diluted 10-fold in DMEM supplemented with streptomycin-penicillin, and 1 μg/ml of TPCK trypsin, followed by inoculation into pre-washed MDCK plates for determining viral titers. The plates containing the inoculated cell cultures were then incubated at 33^o^C for 5 days. The traditional hemagglutination assay (HA) using 1% Turkey RBC (Lampire Biological Laboratories, Pipersville, PA, USA) was used for endpoint measurement of viral titers. Viral TCID_50_/ml (for nasal washes) or TCID_50_/gram (for tissues) were calculated using Reed and Muench method as described previously [79].

### Hemagglutination inhibition (HI) assay

The hemagglutination inhibition (HI) assay was conducted following the guidelines provided in the WHO standard manual [80]. Initially, the pre-challenged and post-challenged sera were pre-treated with receptor destroying enzyme (RDE-II; Hardy Diagnostics, reference number: 370013) by incubating overnight at 37^o^C. Subsequently, the enzyme was inactivated by incubating samples at 56^o^C for 30 minutes. Two-fold serial dilutions of the sera were then incubated with 4 HA units of swine D/OK (D/swine/Oklahoma/1334/2011) or bovine D/660 (D/bovine/Oklahoma/660/2013) virus. Following the serum-virus incubation, 1% Turkey red blood cells (RBCs) were added. Physiological saline and serum controls, as well as virus back titration, also were performed for validation. HI titers were expressed as the reciprocal of the highest dilution of serum that gave complete absence of hemagglutination. All samples were assayed in three separate experiments with each sample analyzed in duplicate and the mean antibody titers were calculated from these triplicate data.

### Histopathology and RNAscope-based *in-situ* hybridization

Samples from the respiratory tract (nasal turbinate, soft palate, trachea, and lungs) and intestinal tract (large and small intestine) were preserved in 10% neutral buffered formalin followed by subsequently embedding in paraffin wax. Thin tissue sections were prepared and underwent staining with hematoxylin and eosin (H&E) for histopathological analysis. The presence of IDV genome in the respiratory and intestinal tissues was identified by RNAscope-based *in-situ* hybridization (ISH) protocol [81], involving the negative-sense RNA probe specific to the polymerase basic 1 (PB1) segment of IDV. Briefly, following the deparaffinization and rehydration, tissue sections were processed for antigen retrieval by boiling in RNAscope Target Retrieval Reagent solution for 15 minutes and treatment with RNAscope Protease Plus at 40°C for 15 minutes. For IDV RNA genome detection, tissue sections were hybridized with IDV antisense PB1 probe (Advanced Cell Diagnostics, Catalog number: 441331) at 40°C for 2 hours. Signals were detected and amplified using the RNAscope 2.5 HD assay-RED kit.

### Identification of IDV-infected *in vivo* cell types and quantification of viral RNA (vRNA) signals

The combination of RNAscope *in situ* hybridization with immunohistochemical staining (IHCS) was used to identify the type of respiratory and intestinal epithelial cells that were infected by IDV. Following the RNAscope, tissue sections were immediately subjected to IHCS. After blocking with a buffer containing 5% bovine serum albumin, sections were incubated overnight at 4°C with an anti-rabbit pan-cytokeratin antibody (Catalog number: ab308262, Abcam, Waltham, MA, USA). Antibody staining signals were visualized using DAB and the Envision GI2 Double stain System Rabbit/Mouse kit (Catalog number: K5361, Agilent Dako, USA), followed by counterstaining with hematoxylin. Digitized images of tissue sections were analyzed using Aperio’s Spectrum Plus analysis program. The frequency of vRNA-positive cells was manually quantified.

### Sialic acid profiling of ferret lung tissue

Lung tissue was homogenized by passing through 26-gauge syringe in high salt buffer (2 M NaCl, 5 mM EDTA) followed by probe sonication for 2 min (10 seconds ON, 10 seconds OFF). Sample was ultracentrifuged at 90000 x g for 15 min at 4^o^C and the resulting pellets were re-suspended in sodium carbonate buffer (0.1 M Na_2_CO_3_, 1 mM EDTA, pH 11.3). After incubation in ice for 30 min, sample was ultracentrifuged at 90,000 x g for 90 min at 4^o^C, the resulting protein pellets were dissolved in lysis buffer (8 M urea, 1 M NaCl, 4% CHAPS, 100 mM DTT, 200 mM Tris-HCl, pH 8.0). Protein samples were then reduced and alkylated with DTT and Iodoacetamide respectively. Reduced protein samples were precipitated using chloroform:methanol:water (1:4:3 by volume) and then protein amount were normalized using BCA protein quantification (Bicinchoninic Acid Assay) [82]. The samples were centrifuged, and the upper layer was removed. An additional equal volume (400 μL) of methanol was then added and samples were centrifuged again. The methanol layer was removed, and protein pellets were then digested. Equal amounts of protein samples were subjected to PNGase F digestion at 37^o^C for 16 h followed by separating N-glycan using a centrifugal filter of 10 kDa MW. De-N-glycosylated proteins were further digested with trypsin (1:20 ratio) at 37^o^C for 16 hours. To release sialic acid species from both N-glycan samples and de-N-Glycosylated peptides, samples were incubated with α-Neuraminidase (3,6,8,9 specificities, NEB) at 37^o^C for 18 hours followed by acid hydrolysis using 2M acetic acid at 80^o^C for 4 hours. The reference sialic acid and liberated sialic acid from samples were fluorescent labeled using 1,2-diamino-4,5-methylenedioxybenzene dihydrochloride (DMB) at 50^o^C for 2.5 hour. Sialic acids were identified using Waters Acquity UHPLC with FLD detector. Following UHPLC condition were used - Column: Agilent Eclipse Plus C18, 2.2 µm, 4.6 × 100 mm, Mobile Phase: acetonitrile:methanol:water (9:7:84 by volume), flow rate: 0.6 mL/min, temperature: 45ºC, run time: 15 min, detection: excitation λ, 373 nm emission λ, 448 nm. Empower 3 software was used for automatic peak integration and to calculate the peak area. Sialic acid profiling for ferret lung tissue was conducted by complex carbohydrate research center (CCRC), University of Georgia at Athens.

### Viral RNA sequencing

Viral RNAs were extracted from nasal washes and homogenates (clinical samples) as well as cell supernatants obtained after inoculation of samples on MDCK cells (Passage 0) using QIAamp viral RNA mini kit (Qiagen; catalog number 52904). RNAs obtained were subjected to ribosomal RNA depletion and DNA library preparations were generated using Illumina Stranded Total RNA Prep Ligation with Ribo-Zero Plus kit (Illumina; catalog number 20040525). Libraries were sequenced on MiSeq platform using V2 chemistry kits. Adapter trimming was performed on-board, and reads were further analyzed on Galaxy open-source platform (https://usegalaxy.org/; version 24.1.3) [83]. Reads were filtered using fastp (version 0.23.4) (minimum length of 30, Phred score of 30) [84]. Variant calling was performed with SNIPPY (version 4.6.0) [85] by mapping to D/swine/Oklahoma/1334/2011 reference sequence (GenBank: GCF_002867775.1) and D/bovine/Oklahoma/660/2013 HEF segment sequence (GenBank: KF425662.1).

### Structural modeling

The HEF structure from D/swine/Oklahoma/1334/2011 (PDB ID:5E65) was chosen to show locations of the mutations present in the receptor binding domain between D/swine/Oklahoma/1334/2011 and D/Bovine/Oklahoma/660/2013. The structure is presented in the context of binding to Neu5,9Ac_2_ (green). Six mutations in the receptor binding domain of the HEF between two viruses are shown in red.

## Supporting information

S1 Data Primary data used for generation of Figs 2, 3, 4, and 9. This dataset includes ferret nasal wash titers, fecal swab titers, and viral titers from intestinal and lung tissues. Ferret body weight and body temperature measurements collected throughout the study are also included.
(XLSX)

S1 Fig Estimation of viral load using qRT-PCR in ferret tissue samples. A qRT-PCR standard curve was generated using 10-fold serial dilutions of D/OK virus, with Ct values plotted against the log₁₀-transformed TCID₅₀/mL. Standard curve (Ct vs viral titer) was used to estimate viral titers in tissue samples collected from ferrets T620 and T625 at 4 days post-inoculation. Trachea samples from T620 and T625 yielded Ct values of 32.5 and 34.4, corresponding to estimated viral titers of approximately 102.17 and 101.61 TCID₅₀/mL, respectively.
(TIF)
