# Supplementary figures and images for: Differential tissue tropism and transmission efficiency of two dominant influenza D clades with overlapping but distinct receptor binding fine specificities in ferrets
Source: PLoS Pathog. 2025 Sep 15;21(9):e1013493. doi: 10.1371/journal.ppat.1013493 (PMC12435653; doi:10.1371/journal.ppat.1013493)

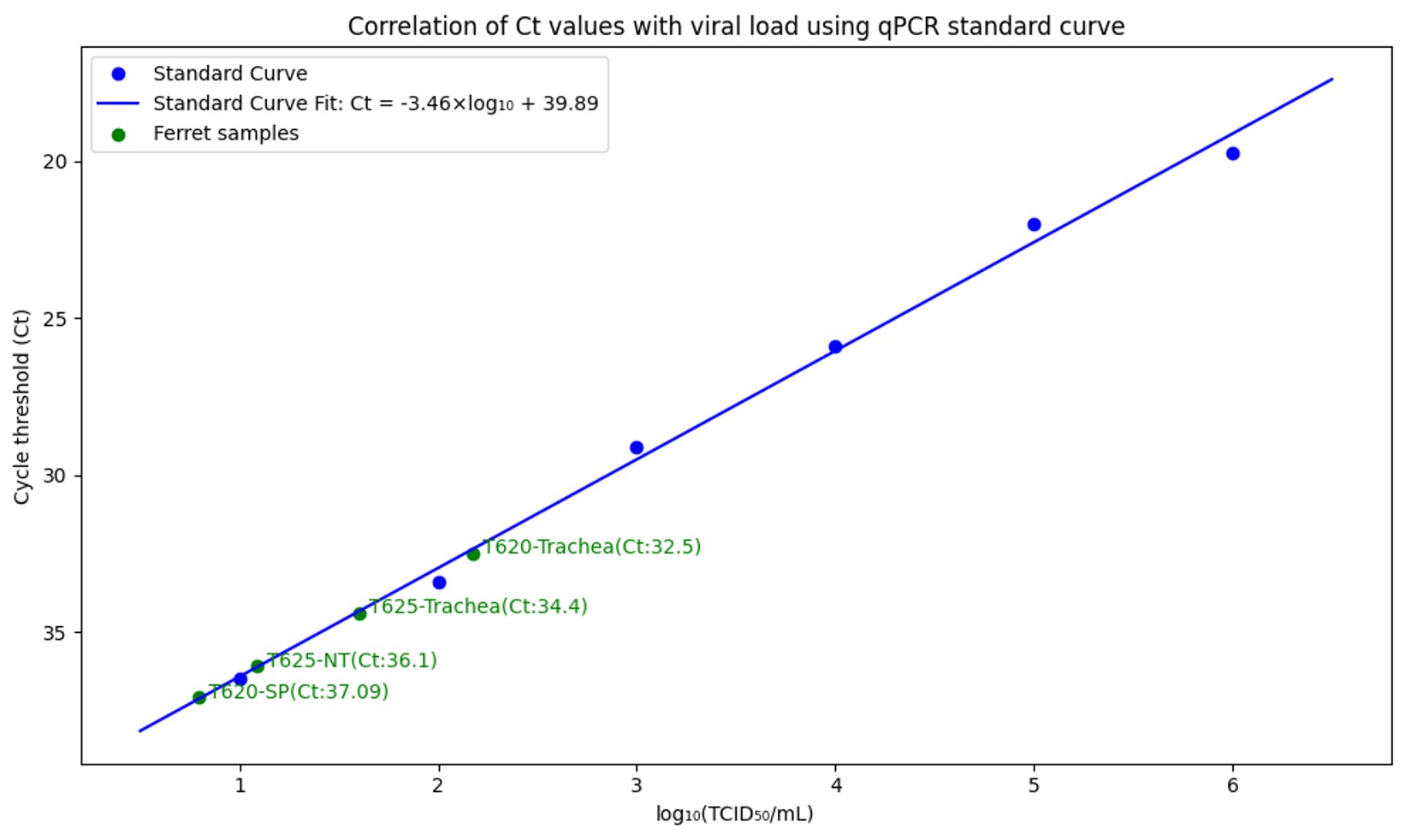

Supplement: S1 Fig — A qRT-PCR standard curve was generated using 10-fold serial dilutions of D/OK virus, with Ct values plotted against the log₁₀-transformed TCID₅₀/mL. Standard curve (Ct vs viral titer) was used to estimate viral titers in tissue samples collected from ferrets T620 and T625 at 4 days post-inoculation. Trachea samples from T620 and T625 yielded Ct values of 32.5 and 34.4, corresponding to estimated viral titers of approximately 102.17 and 101.61 TCID₅₀/mL, respectively. (TIF) [file ppat.1013493.s002.tif]
